# The influence of functional kinematic asymmetry on maximum speed performance in repeated sprints

**DOI:** 10.5114/biolsport.2026.158304

**Published:** 2026-02-23

**Authors:** Mateusz Jopek, Michal Krzysztofik, Dariusz Mroczek, Adam Zajac, Krzysztof Mackala

**Affiliations:** 1 Department of Individual and Team Physical Activities, Wroclaw University of Health and Sport Sciences, al. Ignacego Jana Paderewskiego 35, 51-612 Wrocław, Poland; 2 Institute of Sport Sciences, The Jerzy Kukuczka Academy of Physical Education in Katowice, Mikołowska 72A, 40-065 Katowice, Poland; 3 Department of Biological and Motor Bases of Sports, Wroclaw University of Health and Sport Sciences, al. Ignacego Jana Paderewskiego 35, 51-612 Wrocław, Poland

**Keywords:** Sprinting, Asymmetry, Maximum speed, Kinematics, Biomechanics

## Abstract

The study aimed to determine the influence of functional asymmetry on the kinematic parameters of the 50 m run and its significance in shaping maximum speed in sprinters at various sports levels. The analysis included 18 Polish sprinters (elite: ≤ 10.40 s, sub-elite: ≤ 11.10 s per 100 m) who performed four 50 m runs with 5-minute breaks. Kinematic parameters were recorded using the OptoJumpNEXT system and WittyGate photocells. The fastest and slowest run of each athlete was selected for analysis. The results showed that kinematic asymmetry has a significant impact on sprint performance. Elite sprinters had less asymmetry in stride length, frequency, and ground contact time, which correlated with better results. The key findings indicate that in the acceleration phase (0–20 m), stride length and contact time symmetry were crucial, while in the maximal speed phase (20–50 m), the symmetry of stride frequency was important. A higher sports level was associated with a more optimized running technique, as evidenced by lower kinematic asymmetry. The results suggest that minimizing kinematic asymmetry may be a crucial factor in optimizing the sprinting technique and enhancing performance, offering practical insights for coaches and athletes and empowering them to make informed decisions in their training programs.

## INTRODUCTION

Sprinting is one of the most important athletic competitions, characterized by extreme demands on strength, speed, and precise running technique [1]. Sprint results depend on several factors, including motor skills, biomechanics of movement, physiological conditions, and start and acceleration techniques [2]. The start and initial meters of the run are crucial for achieving optimal maximum speed, and the quality of execution of these phases determines the final sports result [3].

Sprinting speed is a function of two main kinematic parameters: stride length and stride frequency [4]. Efficient speed generation requires an optimal combination of these variables, which depends on individual motor skills and running technique. Studies indicate that increased stride length can lead to a decrease in frequency, so finding a balance between these parameters is crucial for achieving maximum speed [5]. Another factor influencing sprinters’ performance is the generation of ground reaction forces. The vertical and horizontal forces that the athlete transfers to the ground during running have a decisive impact on acceleration and maintaining speed [6]. Optimizing the start technique and acceleration phase enables the maximum utilization of the athlete’s strength potential [7].

In recent years, attention has increasingly turned to the role of functional asymmetry, particularly kinematic asymmetry between limbs, as a potential limiting factor in sprint performance. Although small asymmetries are natural in human movement, emerging evidence suggests that excessive or unaddressed asymmetry may compromise sprinting efficiency, increase energy cost, and elevate injury risk [8–9]. Functional asymmetry, defined as the difference in kinematic and kinetic parameters between the right and left limbs, can significantly impact sprint performance [10]. High asymmetry can lead to inefficient running techniques, increased energy expenditure, and a higher risk of injury [11]. Studies indicate that kinetic asymmetry, manifested by differences in ground reaction force and power generation between limbs, can limit an athlete’s ability to achieve optimal movement dynamics [9]. Similarly, kinematic asymmetry—concerning stride length, take-off angle, and hip angle—affects an athlete’s running balance and stability during acceleration and reaching maximum speed [12]. Both strength and movement asymmetry can result from the dominance of one limb and natural anatomical differences in athletes. However, asymmetry above 10% in specific parameters may negatively affect sports performance, and monitoring and reducing it through targeted training are key elements in preparing sprinters [13]. This study builds on this existing research by specifically analyzing functional kinematic asymmetry across different phases of sprinting in elite vs. sub-elite athletes.

Despite the potential significance of asymmetry, the biomechanical community still lacks a consensus on the extent to which kinematic asymmetry affects sprint performance, particularly in relation to specific phases of sprinting. While prior research has primarily focused on general asymmetry or kinetic aspects (e.g., ground reaction forces), fewer studies have specifically analyzed functional kinematic asymmetry across different phases of sprinting in elite vs. sub-elite athletes. This study aims to address that gap, utilizing highresolution optical systems to assess symmetry during a 50-meter sprint, split into acceleration and maximum velocity phases. Additionally, 3D analysis of movement biomechanics or EMG enables precise diagnosis of asymmetry and the selection of appropriate training strategies [14]. These strategies must focus on exercises that strengthen the weaker limb, improve neuromuscular coordination, and increase the symmetry of the movement pattern.

The main goal of this work is to determine whether and to what extent functional (kinematic) asymmetry is present during 50-meter sprints in sprinters of varying levels. We hypothesize that elite athletes will display lower asymmetry indices, particularly in stride frequency, contact time, and step length, and that these asymmetries will correlate negatively with sprint performance in both the acceleration and maximal speed phases.

## MATERIALS AND METHODS

### Participants

Eighteen well-trained sprinters (ages 17–26 years) from the Polish National Senior and Junior Team (ranging from 100 to 200 m) participated in the study. The participants were divided into two groups: junior (sub-elite: body mass 67.56 ± 6.9 kg, body height 1.78 ± 0.1 m, BMI 21.19 ± 1.2) and senior (elite: body mass 76.72 ± 8.0 kg, body height 1.82 ± 0.1 m, BMI 23.22 ± 1.1). The classification was based on age and personal bests over a distance of 100 m (elite: ≤ 10.40 s; sub-elite: ≤ 11.10 s). The tests require participants to have four to five years of consistent sprint training experience. Each participant had medical clearance to train and reported no injuries that could affect their ability to perform maximumvelocity sprints. All individuals had completed at least six months of sprint-specific strength, speed, and plyometric training before enrollment. Informed written consent was obtained after the participant was thoroughly informed about the protocol and procedures. Additionally, parental consent was secured for those under eighteen. The Bioethics Committee for Scientific Research (3/2021) approved the protocol at the Jerzy Kukuczka Academy of Physical Education. The study was conducted in accordance with the ethical standards outlined in the Declaration of Helsinki (2013).

### Study Design

The experiment was meticulously designed and conducted in January during the national sprint team training camp in the pre-competition period. This allowed for obtaining optimal conditions for assessing the athletes’ readiness for indoor competitions. The research occurred in the Central Olympic Preparation Center athletics hall in Spała. All sprints were conducted under identical indoor conditions, with controlled lighting, surface consistency, temperature (~20°C), and footwear. The experiment lasted two days: on the first day, measurements were taken with the junior group (sub-elite), and on the second day, the senior group (elite) was measured. The 50-meter distance was selected to capture both the acceleration and early peak-speed phases of the sprint, consistent with testing protocols used during pre-competition periods. A standing high start was used, reflecting common sprint training conditions where athletes emphasize maximal acceleration mechanics without the variability introduced by block starts or start reaction time. The OptoJump Next-Microgate sensor measurement system (Optojump, Bolzano, Italy) was used to calculate the essential kinematic variables of sprinting. Additionally, each athlete underwent basic anthropometric measurements (body height and body mass).

### Experimental Session

**FIG. 1:**
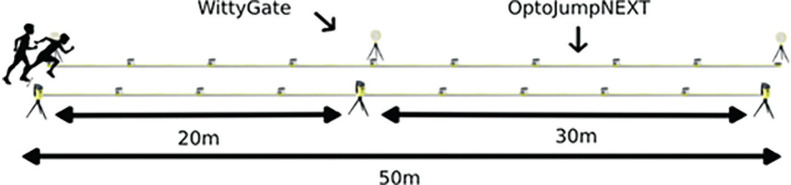
Diagram of the measurement device setup during the 50-meter race.

The OptoJump-Microgate optical measurement system (OptoJump Next, Microgate, Bolzano, Italy) and six Witty photocell gates were applied to an indoor athletics track for four 50-m sprints. The Witty photocells were positioned at the start line, at 20 m, and at the finish line (50 m) (Figure 1). The kinematic variables of each running stride — stride length, stride frequency, ground contact time, flight time, single step time, and stride velocity — were measured using the OptoJump Next system (software version 1.14.0). The OptoJump Next photoelectric system consists of pairs of transmitting (TX) and receiving (RX) bars, each measuring 1 m in length. To record kinematic data along the entire 50 m section of the track, fifty TX and fifty RX bars were connected in series and positioned opposite each other, forming a continuous 50 m optical measurement corridor. The bars were placed across the full width of the running lane, ensuring that each foot contact interrupted the infrared light beams. Each bar emits and receives invisible infrared LED light beams with a spatial resolution of approximately 1 cm. Every foot strike interrupts the light transmission and is detected with a temporal accuracy of 1 ms (sampling rate = 1000 Hz). The system operates contact-free and allows precise detection of contact and flight phases during sprinting. The combination of OptoJump Next and Witty systems enabled the simultaneous collection of kinematic and temporal data throughout the sprint trials.

The sprinters performed four individual sprints, starting from a semi-crouch position. This start aimed to achieve smooth and fast acceleration to reach maximum running velocity. The competitors manage the five-minute rest periods between particular repetitions. Each athlete completed a standardized warm-up and baseline performance assessment, which included two 40-m accelerations at 85–90% of maximum intensity. Both the fastest and slowest sprint attempts per athlete were selected for detailed analysis. This approach allowed evaluation of both maximal capability and variability in movement symmetry, providing insights into how kinematic asymmetry behaves under different performance intensities.

### Statistical Analysis

The following methods were used for statistical analysis: Shapiro-Wilk test to assess normality of distribution, Student’s t-test for independent samples, univariate analysis of variance (ANOVA), and Bonferroni post-hoc test. To assess symmetry of kinematic parameters, the symmetry index (SI) was used, calculated according to the formula described by Vagenas and Hoshizaki [15]:

$$SI=\frac{\left(L-R\right)}{0,5*(L+R)}100\%$$

The findings of this study are significant and provide valuable insights into sprint biomechanics and performance analysis. The asymmetry data were calculated as a percentage of asymmetry, using the absolute difference divided by the mean of the limbs. Before calculating asymmetry, the data were averaged for all complete steps of each limb for the full distance and each of the running phases. In addition, Cohen’s d index was calculated to determine the effect size, and a backward stepwise regression model was built to describe the relationships between variables. Partitioning Around Medoids (PAM) clustering analysis was also performed, enabling the identification of homogeneous groups within the dataset. All analyses were performed at a significance level of p < 0.05, and significant results are marked in bold in the tables.

## RESULTS

The basic anthropometric characteristics and personal sprint records of elite and sub-elite sprinters differed significantly. The elite group was considerably older (+4.7 years), heavier (+9.2 kg), and had a higher BMI (+2.03; all p < 0.05), which likely reflects greater biological maturity and training experience. Sprint performance was also significantly better in the elite group, with faster times in both the 60 m (Δ = 0.38 s, p = 0.0001) and 100 m (Δ = 0.46 s, p < 0.0001) events. Interestingly, no significant difference in body height was observed (p = 0.30), suggesting that sprinting performance at this level may depend more on neuromuscular and biomechanical factors than on stature alone.

Table 1 summarizes the kinematic parameters recorded during the slowest and fastest 50-meter sprints of each athlete, comparing elite and sub-elite groups. Elite sprinters achieved significantly faster 50 m times in both trials (Δ ≈ 0.20 s, p < 0.01), which was associated with higher step frequency (+0.30 Hz) and shorter ground contact time (-0.011 s). As a result, elite athletes displayed a higher single-step velocity (~+0.34 m/s, p < 0.001) and shorter step duration. These findings confirm that technical execution, particularly minimizing ground contact time and maximizing turnover, plays a critical role in sprint performance at the elite level. From a coaching perspective, training strategies that target contact efficiency and rhythm may be particularly beneficial for sub-elite sprinters aiming to progress.

**TABLE 1:** Characteristics of kinematic parameters of the slowest and fastest 50 m run, divided into elite and sub-elite, p < 0.05

Variables		*n*	$\bar{\text{x}}$	s	v	Confidence		t	*p*

						-95%	-95%		
50 m time* [s]	Elite	9	5.427	0.10	1.84	5.35	5.50	**-3.71**	**0.0019**
	Sub-elite	9	5.627	0.13	2.31	5.53	5.73		
50 m time** [s]	Elite	9	5.351	0.11	2.06	5.27	5.44	**-3.49**	**0.0030**
	Sub-elite	9	5.544	0.12	2.16	5.45	5.64		
Step frequency * [Hz]	Elite	9	4.781	0.13	2.72	4.68	4.88	**2.93**	**0.0097**
	Sub-elite	9	4.472	0.29	6.48	4.25	4.69		
Step frequency ** [Hz]	Elite	9	4.784	0.14	2.93	4.68	4.89	**3.03**	**0.0080**
	Sub-elite	9	4.484	0.26	5.80	4.28	4.69		
Ground contact time* [s]	Elite	9	0.105	0.01	9.52	0.10	0.11	**-2.57**	**0.0204**
	Sub-elite	9	0.116	0.01	8.62	0.11	0.12		
Ground contact time** [s]	Elite	9	0.104	0.01	9.62	0.10	0.11	**-2.72**	**0.0151**
	Sub-elite	9	0.115	0.01	8.70	0.11	0.12		
Single step time* [s]	Elite	9	0.210	0.01	4.76	0.21	0.21	**-2.90**	**0.0105**
	Sub-elite	9	0.225	0.01	4.44	0.21	0.24		
Single step time** [s]	Elite	9	0.209	0.01	4.78	0.20	0.21	**-3.07**	**0.0073**
	Sub-elite	9	0.224	0.01	4.46	0.21	0.23		
Single step velocity* [m/s]	Elite	9	9.183	0.14	1.84	9.08	9.29	**4.66**	**0.0003**
	Sub-elite	9	8.807	0.20	2.31	8.65	8.96		
Single step velocity** [m/s]	Elite	9	9.208	0.11	2.06	9.13	9.29	**4.75**	**0.0002**
	Sub-elite	9	8.874	0.18	2.16	8.73	9.01		

Note: n – number of participants; $\bar{\text{x}}$ – mean; s – standard deviation; v – coefficient of variation; t – t-statistic; p – significance level; Hz – hertz; m/s – meters per second; s – seconds;

* – slow trial;

** – fast trial.

Table 2 shows the symmetry indices (SI) for selected kinematic parameters in elite and sub-elite sprinters during both slow and fast trials. Although no statistically significant differences were observed (p > 0.05), several parameters showed moderate to large effect sizes, notably step length (d = -0.87) and step frequency (d = -0.74) in slow sprints. These values suggest that elite sprinters exhibit more symmetrical movement patterns, especially in variables directly linked to stride mechanics. Even in the absence of statistical significance, such trends may indicate functionally relevant asymmetries that affect performance. This supports the growing body of literature recommending regular monitoring of inter-limb differences, particularly in developing athletes. In practice, these asymmetries could help coaches identify subtle inefficiencies or imbalances before they manifest as performance plateaus or injuries.

**TABLE 2:** Characteristics of the symmetry index of the kinematic parameters of the slowest and fastest 50-meter run, divided into: elite and sub-elite, p < 0.05

Variables		*n*	$\bar{\text{x}}$	s	v	Confidence		t	*p*	*d*

						-95%	-95%			
SI Step length* [%]	Elite	9	2.091	1.41	67.43	1.01	3.17	-1.76	0.1029	**-0.87**
	Sub-elite	9	3.821	2.59	67.78	1.83	5.81			

SI Step length ** [%]	Elite	9	1.930	1.31	67.88	0.92	2.94	-1.31	0.2144	**-0.66**
	Sub-elite	9	3.264	2.75		1.15	5.38			

SI Step frequency* [%]	Elite	9	1.347	1.38		0.29	2.40	-1.52	0.1525	**-0.74**
	Sub-elite	9	2.710	2.31	85.24	0.93	4.49			

SI Step frequency** [%]	Elite	9	1.880	1.51	80.32	0.72	3.04	-1.24	0.2413	**-0.62**
	Sub-elite	9	3.359	3.25	96.75	0.86	5.86			

SI Flying phase * [%]	Elite	9	3.514	2.45	69.72	1.63	5.40	-1.07	0.3044	**-0.52**
	Sub-elite	9	5.199	4.05	77.90	2.09	8.31			

SI Flying phase ** [%]	Elite	9	3.518	3.51	99.77	0.82	6.21	-1.51	0.1522	**-0.71**
	Sub-elite	9	6.328	4.36		2.98	9.68			

SI Single step time* [%]	Elite	9	1.295	1.30		0.30	2.29	-1.12	0.2816	**-0.53**
	Sub-elite	9	1.922	1.08	56.19	1.09	2.75			

Note: SI – symmetry index; n – number of participants; $\bar{\text{x}}$– mean; s – standard deviation; v – coefficient of variation; t – t-statistic; p – significance level; d – Cohen’s d (effect size); % – percentage;

* – slow trial;

** – fast trial.

For a better understanding of the course of changes in kinematic parameters, the 50 m sprint was divided into two phases: the first 20 m represents the acceleration phase, and the last 30 m represents the maximum speed phase. The present stepwise regression models (Tables 3 and 4) identify which symmetry indices (SIs) significantly predict sprint time during the 20 m acceleration phase. Among elite sprinters during slow trials, SI values for step length, step frequency, contact time, and step speed all significantly influenced sprint time (R^2^ = 0.95, p < 0.01). Notably, greater asymmetry in step execution speed negatively impacted performance, while more symmetrical step length, frequency, and contact time were associated with better times. In sub-elite athletes, only step length and contact time asymmetries were significant predictors, suggesting a more limited technical profile. Interestingly, asymmetry in contact time showed an adverse effect, indicating that unequal push-off timing between legs may directly reduce early acceleration efficiency. For elite athletes during fast trials, only contact time asymmetry remained significant (R^2^ = 0.57), highlighting its central role at higher intensities. In contrast, no significant predictors emerged for sub-elite athletes in fast trials, possibly due to greater variability or inconsistent technical execution.

**TABLE 3:** Stepwise regression results for the symmetry indices of kinematic parameters over distances of 20 (starting acceleration) for the elite and sub-elite, p < 0.05

Variables	Elite 20 m				Variables	Sub-elite 20 m			

Slow trial	b	p	R^2^	p		b	p	R^2^	p
Intercept	**2.3423**	**0.0000**	**0.9544**	**0.0015**	Intercept	**2.5858**	**0.0000**	**0.8155**	**0.0088**
SI Step length	**0.0321**	**0.0007**			SI Step length	**0.0173**	**0.0278**		
SI Step frequency	**0.1079**	**0.0003**			SI Ground contact time	**-0.0196**	**0.0113**		
SI Ground contact time	**0.0191**	**0.0004**			SI Flying phase	**-0.0070**	**0.0126**		
SI Step time	**-0.0810**	**0.0003**							
Fast trial	b	p	R^2^	p	none	b	p	R^2^	p
Intercept	**2.2049**	**0.0000**	**0.5686**	**0.0115**		no significance			

Note: b – regression coefficient; p – significance level; R^2^ – coefficient of determination; SI – symmetry index.

**TABLE 4:** Stepwise regression results for the symmetry indices of kinematic parameters over distances of 30 meters (maximum speed) for the elite and sub-elite, p < 0.05

Variables	Elite 30 m				Variables	Sub-elite 30 m			

Slow trial	b	p	R^2^	p		b	p	R^2^	p
none	no significance				Intercept	**3.0029**	**0.0000**	**0.9393**	**0.0114**
					SI Step length	**-0.0430**	**0.0136**		
					SI Step frequency	**-0.0370**	**0.0331**		
					SI Ground contact time	**0.0628**	**0.0025**		
					SI Flying phase	**0.0389**	**0.0053**		
					SI Step velocity	**-0.0370**	**0.0299**		

Fast trial	b	p	R^2^	p	b	p	R^2^	p

Intercept	**2.9151**	**0.0000**	**0.5485**	**0.0388**	Intercept	**2.9914**	**0.0000**	**0.3718**	**0.0478**
SI Step length	**-0.0164**	**0.0183**			SI Ground contact time	**0.0247**	**0.0478**		
SI Step frequency	**0.0167**	**0.0238**							

Note: b – regression coefficient; p – significance level; R^2^ – coefficient of determination; SI – symmetry index.

**TABLE 5:** PAM profile analysis of symmetry indices of kinematic parameters at distances of 20 (starting acceleration) and 30 meters (maximum speed) for Elite and Sub-Elite, p < 0.05.

Sprint	Parallelism						Horizontality				Flatnes			

	Test	Statistic	F	n.df1	d.df1	p	SS	MS	F	p	F	df1	df2	p
20 m slow	Wilks	0.5345	2.09	5	12	0.1371	0.01	0.01	0.0	0.9670	4.80	5	12	**0.0121**
20 m fast	Wilks	0.7060	1.00	5	12	0.4584	6.95	6.95	2.0	0.1800	7.10	5	12	**0.0026**
30 m slow	Wilks	0.7440	0.83	5	12	0.5550	4.53	4.53	1.2	0.2930	4.54	5	12	**0.0148**
30 m fast	Wilks	0.6536	1.27	5	12	0.3375	0.23	0.23	0.1	0.7650	2.78	5	12	0.0678

Note: SS – sum of squares; MS – mean square; F – Fisher’s F statistic; df1, df2 – degrees of freedom; p – significance level; Λ – Wilks’ lambda.

For a deeper understanding of the kinematic symmetry problem, the PAM profile analysis and its interpretation were undertaken (Table 5). For the 20 m slow sprint, Wilks’ Λ = 0.5345, F = 2.09, p = 0.1371, indicated no significant differences between groups. However, the interaction was significant (F = 4.80, p = 0.0121), Wilks’ lambda. suggesting variability driven by interdependent variables. Although the profiles were not parallel, the differences were minor. Significant flatness differences reflected variability across conditions. In turn, the 20 m fast sprint indicates that Wilks’ Λ = 0.7060, F = 1.00, p = 0.4584, also showed no main effect. Still, the interaction was significant (F = 7.10, p = 0.0026), indicating group-specific differences at higher speeds. Profiles remained parallel, with similar overall results, though flatness differences highlighted variability in performance under fast conditions.

For the 30 m slow sprint, Wilks’ Λ = 0.7440, F = 0.83, p = 0.5550, suggested no parallelism effect. However, group means differed significantly (p = 0.0293), and flatness was significant (F = 4.54, p = 0.0148), showing that kinematic variables varied considerably within groups. In the case of the 30 m fast sprint, Wilks’ Λ = 0.6536, F = 1.27, p = 0.3375, indicated no significant effects. The interaction (F = 2.78, p = 0.0678) and flatness differences approached significance, suggesting potential trends in group variability that warrant further study.

## DISCUSSION

This study, which delved into the impact of functional kinematic asymmetry on sprint performance during various phases of a 50-meter run in athletes of varying skill levels, has yielded significant findings. Our research confirms that elite sprinters outperform subelite athletes in both kinematic performance and movement symmetry, aligning with earlier studies [16–19]. Key performance differentiators, such as higher step frequency and shorter ground contact time, have been further validated by studies conducted by Delecluse [20], Muller et al. [21] Bell [22]. The preference for more symmetrical limb coordination among the elite, as suggested by our findings, is in line with earlier studies by Mero et al. [2], Meyers et al. [23], Bezodis et al. [7], Miyashiro et al. [24], Bissas et al. [16], and D’Hondt et al. [25].

### 50-meter sprint

Elite athletes completed the 50-meter sprint significantly faster in both slow and fast trials. The difference was 0.20 s in slow sprinting and 0.193 s in fast sprinting, which is statistically significant (p < 0.05). These differences were primarily driven by a higher step frequency (slow trial: 0.309 steps/s and fast trial: 0.30 steps/s, p < 0.05), a key factor in sprint performance, rather than stride length, aligning with findings by Sunaryadi [26]. Although stride length was not statistically different, its symmetry index showed significant effects favoring elite athletes (-0.87 and -0.66, respectively, for slow and fast sprints). Similarly, moderate effects were found for stride frequency symmetry. Nevertheless, the strong correlation between stride length and frequency in the final result indicates that these variables are essential in influencing running time [4, 27]. This suggests that elite athletes maintain better interlimb coordination over the whole sprint, even when asymmetry is subtle. In turn, the ground contact time was consistently shorter in elites (a difference of 0.011 s), enabling a quicker transition to flight, which in turn reduces the single-step time and increases the step speed [2, 6]. This also results in a higher step speed (0.376 m/s and 0.334 m/s, respectively, for slow and fast trials). It should be assumed that elite sprinters possess a stronger ability to generate higher speeds due to the optimal combination of step frequency, which is dependent on the efficiency of ground contact time [28–31].

### 20 m sprint – acceleration phase

The acceleration phase is critical for reaching peak sprint speed [2]. Elite sprinters showed significantly faster 20 m times (0.057 s in slow runs; 0.076 s in fast runs), reflecting more effective starts and push-offs [7]. Backward stepwise regression analysis revealed that step length symmetry positively influenced performance in both elite and sub-elite athletes, although the exact impact of asymmetry remains unclear [3]. Faster sprinters generally showed less step length asymmetry [32]. Elite athletes also displayed higher stride frequencies (+0.276 steps/s in slow runs, +0.291 steps/s in fast runs), a key factor in acceleration [33]. Regression analysis confirmed its positive effect on elite athletes, suggesting that greater stride frequency symmetry contributes to a more efficient start and better performance. Greater stride frequency symmetry further improved efficiency [34], although this was not evident in younger athletes, specifically boys aged 11–16 years [23]. Elite sprinters had shorter ground contact and flight times, which enabled faster step transitions into the flight phase and reduced step duration [2, 7]. Contact time symmetry is beneficial for acceleration [3]. Regression results support our findings: in fast runs, contact time symmetry was the only significant predictor of elite performance. In turn, excessive asymmetry has been found to impair performance [7], as minimizing imbalances ensures more powerful and coordinated push-offs, thereby boosting acceleration and top speed. However, Bissas et al. [16] found that the effect of contact time symmetry is minimal, noting that mechanical asymmetry is generally low among elites and varies individually. Flight phase asymmetry hindered sub-elite performance, while balanced limb action improved acceleration [3]. Elite athletes also achieved higher step speeds (+0.264 m/s in slow runs and +0.224 m/s in fast runs), a decisive factor in later speed [29, 35]. Although step speed symmetry was not statistically significant, moderate effects suggested more efficiency among elites. Conversely, sub-elites suffered from step speed asymmetry [24]. However, previous studies have suggested that maintaining symmetrical step speeds improves coordination and efficiency, thereby supporting the development of higher sprinting velocities.

### 30 m sprint – maximum speed phase

The final 30 meters represents the maximum speed phase, where athletes reach peak velocity, regardless of their performance level [1]. Elite sprinters ran significantly faster (0.143 s in slow runs and 0.116 s in fast runs), confirming that higher-level athletes can maintain top speed for a more extended period [2]. This ability is strongly linked to stride frequency, which was higher among elites (+0.348 steps/s in slow, +0.305 in fast runs). According to this, Ae [36] indicated that the ability to maintain maximum speed depends on achieving maximum stride frequency; however, in elite athletes, both parameters increase simultaneously. Regression analysis confirmed that stride frequency is key for both sub-elites (in slow runs) and elites (in fast runs), while asymmetries may represent individual adaptations, such as those observed in sprinters of different performance levels [5]. However, these asymmetries did not necessarily limit performance; instead, they reflected individual adaptations and training strategies used to maximize speed potential through optimized sprint technique.

Stride length showed no significant differences between groups, but it was more symmetrical in the elite group. In fast runs, an effect size of –0.66 indicated that stride length symmetry supports stable speed maintenance. This was confirmed by Babic et al. [37]. Regression results also revealed that high stride length asymmetry hindered performance in slow runs for sub-elite athletes, highlighting the importance of symmetry in elite fast runs. D’Hondt et al. [25] emphasized in their study of sprint starts that stride length asymmetry is undesirable for efficiency, not only during acceleration but also at maximum speed. He emphasized the need for larger, high-quality studies to fully understand these relationships and their implications for sprint performance. Elite sprinters also displayed shorter contact times and flight phases, which enhance power generation [30–31]. Although some studies suggest a negative link between contact time symmetry and efficiency [38], He studied elite French 100 m sprinters. He found a negative correlation between contact time symmetry and sprint efficiency, which contrasts with earlier findings. This discrepancy may be due to longer distances (100 m) complicating asymmetry analysis. Nonetheless, high-level sprinters appear to be better able to synchronize stride length and frequency to optimize ground contact time. Such coordination helps achieve higher step speeds, as previously confirmed by Delecluse et al. [20] and Murphy et al. [28]. Overall, synchronization of stride length, frequency, and contact time remains crucial. Elites also exhibited more symmetrical flight phases, which improve stability and efficiency [7]. However, Haugen et al. [39] cautioned that imbalances in air time can disrupt mechanics, reduce efficiency, and increase the risk of injury. Step speed was again higher in elites, with asymmetry reducing efficiency among sub-elites [40].claimed that reducing asymmetry enhances sprint efficiency. Ultimately, efficiency arises from a balance between motor potential and technical skill, underscoring the importance of executing symmetrical steps at maximum speed.

### Limitations

The experiment revealed several critical limitations that must be addressed in future research to enhance our understanding of kinematic asymmetry in sprinting. By tackling these issues, we can gather more comprehensive data and achieve more accurate interpretations of performance. For instance, using a standing start, while beneficial for speed training, lacked the rigor of a traditional block start. This method engages different muscle groups and results in higher initial velocities. Future research could compare the two starting methods to understand their impact on asymmetry. Additionally, the sprinters began the race voluntarily, without a command or starting signal, which eliminated the measurement of their reaction times and could distort asymmetry assessments in the initial race phase. Future studies could use a standardized starting signal to measure reaction times and their impact on asymmetry during initial acceleration. The measuring equipment was limited to 50 meters, meaning sprinters may not have reached their maximum speed, which could have led to different movement patterns compared to longer distances, such as 60 meters or 100 meters. Future research could use longer distances to understand the impact on asymmetry. Moreover, the study only assessed mass, height, and BMI, neglecting lower limb strength, which is crucial for understanding asymmetry. Future studies could incorporate more detailed measurements, such as limb length and muscle circumference, to provide a richer insight into the physical determinants of asymmetry.

## CONCLUSIONS

Functional (kinematic) asymmetry of the lower limbs is observed during sprinting and has a significant impact on performance results. Studies have shown that elite athletes who achieve better 50 m times exhibit less asymmetry in key kinematic parameters: stride length, stride frequency, and ground contact time. Dividing the 50-meter sprint into two phases significantly differentiated selected kinematic parameters in terms of symmetry and asymmetry. Understanding these differences could lead to more effective training strategies. For instance, during the acceleration phase (the first 20 meters), less asymmetry in stride length and stride frequency resulted in better performance. In the maximum speed phase (the last 30 meters), stride frequency symmetry is crucial for performance. A higher sports level (better experience and technical efficiency), in addition to the result, affects the lower index of asymmetry of kinematic parameters, which is associated with a more optimized performance. As running speed increases, the value of functional symmetry and asymmetry of kinematic parameters in sprinting changes. Sprinters who can maintain greater symmetry at higher speeds achieve better results. Elite athletes show less asymmetry compared to sub-elite athletes.

### Practical implication

These results support the idea that improving lower-limb symmetry—especially in contact time and stride frequency—may enhance acceleration performance, particularly for sub-elite sprinters with less developed motor patterns. Coaches, sports scientists, and athletic trainers who work with sprinters may consider implementing unilateral strength drills and neuromuscular coordination exercises that target timing efficiency in the initial steps of the sprint.
